# Peer assessment to improve medical student’s contributions to team-based projects: randomised controlled trial and qualitative follow-up

**DOI:** 10.1186/s12909-019-1783-8

**Published:** 2019-10-15

**Authors:** Regien Biesma, Mary-Claire Kennedy, Teresa Pawlikowska, Ruairi Brugha, Ronan Conroy, Frank Doyle

**Affiliations:** 1 Department of Epidemiology and Public Health Medicine, Royal College of Surgeons in Ireland (RCSI), Dublin, Ireland; 2 School of Health Care, Faculty of Medicine and Health, Leeds University, Leeds, UK; 3 Health Professions Education Centre, Royal College of Surgeons in Ireland (RCSI), Dublin, Ireland; 4 Centre for Data Management, Royal College of Surgeons in Ireland (RCSI), Dublin, Ireland; 5 Department of Health Psychology, Royal College of Surgeons in Ireland (RCSI), Dublin, Ireland

**Keywords:** Randomised controlled trial, Medical education, Undergraduate, Peer assessment, Teamwork

## Abstract

**Background:**

Medical schools increasingly incorporate teamwork in their curricula but medical students often have a negative perception of team projects, in particular when there is unequal participation. The purpose of this study is to evaluate whether a novel peer evaluation system improves teamwork contributions and reduces the risk of students “free loading”.

**Methods:**

A cluster randomised controlled trial (RCT) with qualitative follow up enrolled 37 teams (*n* = 223 students). Participating teams were randomised to intervention group (19 teams) or control group (18 teams). The validated Comprehensive Assessment Team Member Effectiveness (CATME) tool was used as the outcome measure, and was completed at baseline (week 2) and at the end of the project (week 10). The team contribution subscale was the primary outcome, with other subscales as secondary outcomes. Six focus group discussions were held with students to capture the team’s experiences and perceptions of peer assessment and its effects on team work.

****Results**:**

The results of the RCT showed that there was no difference in team contribution, and other forms of team effectiveness, between intervention and control teams. The focus group discussions highlighted students’ negative attitudes, and lack of implementation of this transparent, points-based peer assessment system, out of fear of future consequences for relationships with peers. The need to assess peers in a transparent way to stimulate open discussion was perceived as threatening by participants. Teams suggested that other peer assessment systems could work such as rewarding additional or floating marks to high performing team members.

**Conclusions:**

Other models of peer assessment need to be developed and tested that are non-threatening and that facilitate early acceptance of this mode of assessment.

## Background

Medical practitioners are expected to work effectively in teams and provide peer feedback to ensure quality of care in clinical practice [1–3]. Consequently, standards and outcomes for medical education and training worldwide require medical students to learn to work effectively in teams and develop reflective skills of self and peers throughout their careers [4–6]. It is therefore important to provide medical students with opportunities to develop and practice teamworking skills. Team learning for medical students is associated with various positive educational outcomes [7, 8]. Consequently, medical school curricula today incorporate active learning modalities to develop such skills. However, many students have a negative perception of team projects which often occur when one or more members of a team do not contribute equal amounts of the work, also known as the free-loader problem [9].

Peer assessment has been proposed as a possible solution to such problems, when teaching staff cannot directly observe each members’ contributions [10, 11]. Peer assessment, grounded in social constructivist theory [12], allows learners to consider and specify the level, volume or quality of work completed by other individuals of equal status in learning or professional terms [13–17]. It may be employed in a variety of educational settings and has been demonstrated to be widely used in the assessment of medical students. The use of peer assessment in medical education has been demonstrated to be advantageous from a number of viewpoints [18]. It encourages students to self-appraise performance and that of others, stimulates educational activities and encourages active participation of students [13, 18, 19]. However, there are concerns cited over the approach to peer assessment and the tools used in such assessments [18, 20, 21].

Perhaps of greatest concern is the peer assessment as a social process and the difficulties this may create for participants [22]. Whilst it has been demonstrated to have positive effects for students, interpersonal relationships and social acceptability should be considered as influential dimensions in the process. Bias may occur when friendships or social interactions influences the approach to peer assessment [23]. This relational effect has been reported elsewhere in the literature on small group behaviour and interactionist theory.

The marking system used to assess peers should also be taken into consideration. In a systematic review about student peer assessment in medical education, 22 different tools used mainly in medical education settings were identified. There was great diversity reported between scoring systems and psychometric characteristics of the tools and no golden standard of peer assessment could be identified [18]. None of the assessment tools identified in the trial resemble that employed in this research.

In this study, we developed and evaluated a novel peer assessment system that was designed to stimulate an open and fair distribution of the available marks for team members and to enable group members to directly address the problem of free-loading. We hypothesized that team contribution and functioning would be higher in teams that used this system in comparison to those without it. To our knowledge, this is the first RCT to assess the effect of a peer-assessment system on team effectiveness among undergraduate medical students. The mixed methods design also permitted us to obtain students’ assessments of peer assessment and intervention implementation.

## Methods

We followed the Consolidated Standards of Reporting Trials (CONSORT) statement for RCT reporting [24].

### Setting of the study

The Royal College of Surgeons in Ireland (RCSI) is characterised by cultural diversity with around 60% of students from the Far East and the Middle East, with less than 20% from Ireland [25, 26]. The study took place during the Population and International Health (PIH) team-based project in semester 3 in the second year of medical undergraduate programme and is compulsory for all students. Over the years several variations of peer assessment have been implemented based on annual student feedback surveys [27]. The current team-based project included a system of peer assessment which requires students to openly discuss and assess team work contributions of peers and achieved by team consensus. Students needed to allocate a fixed overall number of marks which means that if one or more excelling team members were awarded with extra peer marks, this would go at the expense of other (underperforming) team members who would receive reduced marks. While the team-based PIH project was a compulsory part of the module, students were not obliged to take part in the study on this project, and a decision not to participate did not have an effect on their participation in RCSI or future grades. Those who did not want to participate in the study were required to use peer assessment, as this was the usual procedure for the team-based project. All students received project instructions, which require teams to conduct a review of literature on a public health problem and report on teamwork. Students also received training from a librarian on sources of data and searching literature. In addition, teams were asked to keep track of team activities through submitting three team reports during the duration of the project including minutes, team goals, team member roles, team rules and attendance.

### Processes, intervention and control

All second-year medical students enrolled in the module in 2014/2015 were invited to participate in September 2014. Students (*n* = 351) were stratified by gender, nationality and English native speakers in to project teams to create as balanced teams as possible (this is a standard procedure in the team-based project). Randomization was performed with the use of computer-generated random numbers. Consenting students (*n* = 109) were randomly assigned to teams where students would jointly work on a project without the possibility of assessing each other’s performance. The remaining students (*n* = 114) were randomly assigned to teams where an additional component of the team-based project was a novel peer-assessment system.

Teams received detailed instructions to provide assessment to peers as to encourage active participation and equal contribution of students in teams. The peer assessment system had two important features: open feedback and balanced peer marking. First, students had to fill in a form in which they gave detailed feedback on their team members’ contributions to team activities in terms of preparation, contribution, respect for others’ ideas and flexibility. It was essential that teams kept good records of meetings called, of team members’ attendances for each meeting, and could demonstrate the contributions (and lack of contributions) of individual team members.

The peer marking section required students to mark each of the other members of the team. Each team had a fixed number of points to allocate (see Table 1). Students could assign a maximum of 6 marks to each team member. This meant that a total of 24 marks could be assigned in a 6-member team. Students were specifically asked to differentiate some of the ratings depending on how team members contributed to the team. This meant that if one member got a score of 5, another team member must be given a score of 3. The final score was to be agreed by the team. If awarding less than 4 marks to a team member it was essential that the team was able to demonstrate, using evidence, sufficient shortfall in the individual’s contribution to justify the reduced peer mark. Where an individual team member wished to challenge a team decision to award him or her with a low mark, s/he needed to be able to demonstrate, using evidence, that s/he merited a higher peer mark than the other team members had proposed.

### Outcomes

The primary outcome was change in each team members’ contribution to the team, as assessed by the Comprehensive Assessment Team Member Effectiveness (CATME) Likert Short Version tool [14] which describes behaviors typical of various levels of performance in five categories (Contributing to the Team’s Work, Interacting with Teammates, Keeping the Team on Track, Expecting Quality, and Having Relevant Knowledge, Skills, and Abilities). Raters rate each teammate on each item using Likert scales (*strongly dis- agree–strongly agree*). All participating students were asked to complete the CATME at baseline (week 2) and at the end of the project (week 10). This validated questionnaire uses a behaviorally anchored rating scale to measure team-member contributions that are clustered into five broad categories [28]. Secondary outcomes were other CATME subscales: team member interaction, teamwork progress; teamwork quality and having relevant knowledge, skills and abilities. Students allocated to the intervention arm (and those who chose to opt out of the trial) were supplied with detailed instructions on the procedure for peer assessment. Teams were required to submit feedback and allocation of peer marks along with the final project report.

### Sample size

With a median average of 6 students per team, we computed 58 teams conducting the compulsory team-based project. Assuming a response rate of at least 30%, (60 students and 10 groups in each arm), we powered the trial to having 10 teams in each arm. Using the *clustersampi* command in Stata 13.0, and estimating data from Ohland et al., we were powered for a detectable difference of 0.37, assuming the control group mean was 4.59 (SD 0.62), an average of 6 people per team and an intra-class correlation co-efficient rho = 0.05 [28, 29].

### Statistical analysis

Potential demographic differences between intervention and control groups were assessed using t-tests or chi-square χ^2^ as appropriate. Linear regression predicted primary and secondary outcomes at follow-up, controlling for the relevant baseline CATME subscale score. Robust variance estimators were used to account for clustering within teams. Data was analysed as per protocol analysis.

### Focus group discussions

We conducted focus group discussions with six teams to better understand how the peer assessment system contributed to teamwork and to assess the extent to which the intervention had been conducted according to plans and protocols (i.e. implementation). We stratified the teams according to their ultimate team score (high, intermediate and low) and interviewed three teams that participated in the intervention and three control teams. Focus groups were conducted over a 5-day period, and both the focus group facilitator (M-C K) and the participants were unaware of the CATME results. The focus group discussions were recorded on the free download software Audacity® on a portable recording device, and transcribed verbatim for analysis. The transcripts were exported to NVivo Version 10, read repeatedly to reach data immersion and then thematically analysed [30]. Transcripts were firstly coded with themes emerging from the data following organisation and refinement of the codes.

## Results

### Quantitative results

**Fig. 1:**
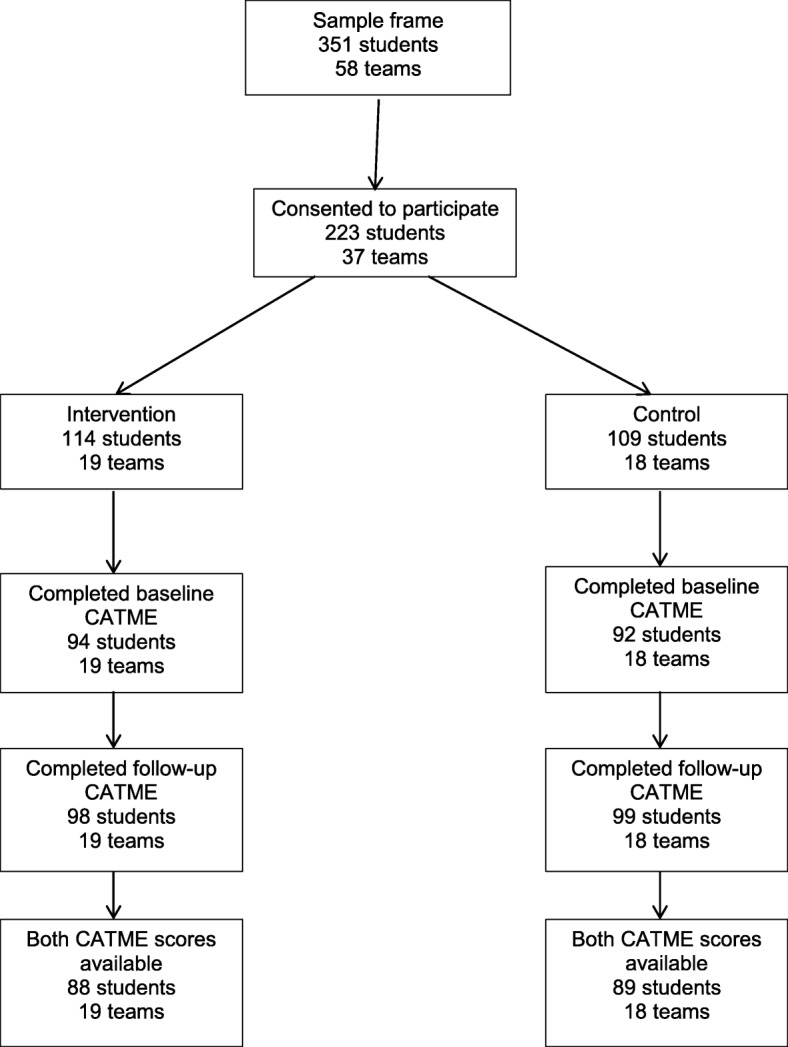
Participant Flowchart

A total of 37 teams (*n* = 223 students) participated in the study and 19 teams were randomised to the peer-assessment, with 18 control teams (see Fig. 1).

About one third of the students were from the Middle East and another one third from South-East Asia while less than 15% of the students were Irish. Age, gender and student region of origin was not associated with participation (all *p*-values>.209), indicating that randomisation was successful. Descriptive statistics are shown in Table 2.

**Table 1:** Balanced, consensus-based peer asessment system

Level team member	Description	Marks^a^
Outstanding	Met all of their own requirements and have demonstrated evidence of leadership and team working skills far beyond what was expected	6
Very good	Met all of their own requirements and covering extra work beyond what was expected	5
Essential	Met all of their own requirements (perhaps after renegotiation of tasks)	4
Adequate	Covered most of the agreed tasks during the team work project	3
Underperforming	Covered some of the agreed tasks	2
Largely underperforming	Failed to meet agreed tasks and who have consistently failed to comply with the set team rules.	1
Free rider	No contribution to the team	0

^a^The overall team mark needs to add up to a total of 24 marks in a 6-member team, 20 marks for a 5-member team and 16 for a 4-member team

**Table 2:** Descriptive statistics in sample of 220 undergraduate students

	Total	Peer assessment	Control	Statistic	*p*-value
Age (mean, SD)	21.3 (1.7)	21.4 (1.9)	21.2 (1.6)	t = −.98	0.32
Women	46.6%	46%	47.2%	x^2^ = .03	0.85
Region of origin					
Ireland/UK	14.6%	15.0%	14.0%	x^2^ = .20	0.99
US/Canada	16.8%	15.9%	17.8%		
Southeast Asia	32.3%	31.9%	32.7%		
Middle East	27.7%	28.3%	27.1%		
Other	8.6%	8.9%	8.4%		

Both in the intervention and control arm, the highest mean scores for the CATME subscales was’ teamwork quality’ while ‘having relevant knowledge, skills and attitudes’ was scored lowest at both baseline and follow up (see Table 3).

**Table 3:** Mean (SD) CATME values at baseline and follow-up

CATME subscale	Peer assessment (intervention)		No peer assessment (controls)	
	Baseline (*n* = 94)	Follow-up (*n* = 98)	Baseline (*n* = 92)	Follow-up (*n* = 99)
Contributing to the team’s work	4.10 (0.73)	4.08 (0.75)	4.02 (0.72)	3.92 (0.83)
Interacting with teammates	4.12 (0.70)	4.09 (0.72)	4.18 (0.62)	4.14 (0.68)
Keeping the team on track	4.06 (0.69)	4.06 (0.78)	4.07 (0.68)	4.04 (0.75)
Expecting quality	4.31 (0.66)	4.33 (0.74)	4.28 (0.66)	4.29 (0.72)
Having relevant KSAs	3.92 (0.81)	4.01 (0.86)	3.89 (0.82)	4.12 (0.71)

Table 4 provides the results of the linear regression, which predicts each outcome by group, controlling for the baseline scale score. There were only small and no statistically significant differences in the means of any of the primary or secondary outcomes between groups.

**Table 4:** Differences in mean team performance outcomes between groups, controlling for baseline scores- linear regression models

	β (95% Confidence interval)	*P*-value
Contributing to the team’s work	0.76 (−0.58 to 2.09)	0.26
Interacting with teammates	−0.31 (−1.92 to 1.31)	0.70
Keeping the team on track	0.41 (−1.01 to 1.83)	0.56
Expecting quality	0.10 (−0.59 to 0.79)	0.78
Having relevant knowledge, skills and abilities	−0.52 (−1.37–0.34)	0.23

### Qualitative results

Three themes were identified in the course of the analysis: anxiety and poor implementation of the intervention, conflicting views whether peer assessment could improve team effectiveness, critical views of the balanced marking system and recommendations for the future (Table 5).

**Table 5:** Themes from qualitative analysis

Anxiety and poor implementation of the intervention	“The group reaction ‘en masse’ was not positive and that it would be better to have the possibility of not being in the peer assessment group … I think I was definitely influenced by the group reaction being concerned about I” (Intervention 3) “Largely it’s political correctness, I’d just probably, I wouldn’t grade that unless it was serious, serious – it would have to be serious …” (Intervention 3) “A couple of my friends had people like that in their group and did nothing. And they were even more stressed because they knew that there was a peer evaluation but the team members wouldn’t vote someone low marks, you know what I mean? So it was time-consuming and more stressful than just not having to mark them. There is tension anyway between you and that person, and how do you tell them that you’re going to give them that grade, it puts even more tension because you have to see them after …’ (Intervention 1) “Maybe if you’re in the position of putting the work together and editing it and you weren’t happy with someone’s work, if there’s no peer evaluation you might be more inclined to tell them that you weren’t happy with how they’re working, just because it works both ways, like as you want them to give you a good mark, so maybe if you’re not happy with how they’re working and you’re not doing peer evaluation, you might not be afraid to kind of tell them like to get their act together or whatever”. (Intervention 2)
Conflicting views whether peer evaluation could improve team effectiveness	**“**Freeloaders regardless of peer evaluation would definitely just coast, regardless. And we’ve seen other groups and we hear about this too, about how peer evaluation or not, there will always be that one girl/guy … who doesn’t want to, who will either say it up front or just be sly about it and then like have someone else do their work for them last minute which is worse in my opinion. But you always have those people, regardless”. (Intervention 1) “Some people actually worked harder but there were some people that just took advantage of the fact we were not marking each other and some people had to pick up the slack then”. (Control 1) “Some people got the feeling it would not make that much of a difference because the whole project is about 17%. One or two marks above or below would not make that much of a difference” (Intervention 3) “If I wanted to work hard, I would have worked hard regardless of the peer assessment... Not because of peer assessment but just because you do not want to seem dumb in front of your friends”. (Intervention 1) “We had people who did their tasks and had no other parts to do during the last days and did not even interact in giving ideas and stuff “(Control 2)
Critical of balanced marking system	“If you think somebody deserves more well then that’s somebody else who deserves less, but not necessarily, we could have all been working at the exact same level but just one person outshines everybody else … it’s like okay well who are we going to take that one point off?” (Control 1) “Say everyone does their part correctly and well, but there is one or two people that do exceptionally well … why should one guy who did as well as the other four, be forced to give up his marks to the other two people that did exceptionally well?” (Control 3)
Recommendations for the future	“If you took an equal from all four [team members] and gave it to the two in a split, then all of them, all the four that did contribute but not as much as the other two, they would still get the same level but the other two would get the points they deserve …” (Control 3) “I think it would be a good idea instead of taking marks away from others, what you can do is you can maybe have a vote … I know it’s out of six, have maybe two or three marks up for grabs and essentially members of the group can, I guess sort of vote for, and it’s unbiased, just vote for who you think should get those extra marks, and I think it would make everyone happy because the people that … didn’t go above and beyond, they’re going to keep their marks and they’re not forced to give their marks to other people, and the people that did go above and beyond, they have a shot at getting those extra marks, so I guess it would benefit both people”. (Control 3)

Overall, intervention teams did not implement peer assessment with the main reason being fears and concerns associated with the possibility of having to mark other team members negatively and the tension this may introduce. The need to assess peers in a transparent way to stimulate open discussion was perceived as threatening by participants. There were also disagreements among the teams on the effect of peer assessment on team effectiveness. For example, teams in the intervention group did not think that peer assessment reduced freeloading, alter their approach to the assignment or how they interacted with team members. In contrast, teams in the control arm were more likely to think that peer assessment could have helped them to improve team work effectiveness, especially when some team members disengaged when they had completed their part of the assignment.

Finally, teams also recommended alternative approaches of peer assessment, such as the implementation of additional or floating marks that were not sacrificed by colleagues. It was proposed that such a system would remove the tension and pressure that currently surrounds the peer marking assessment and also award positive contributions to team performance.

## Discussion

This is the first randomised trial to evaluate a peer assessment intervention during a team-based project in undergraduate medical students. Our data does not confirm the hypothesis that team contribution and functioning is higher in teams that used peer assessment in comparison to those without it, mainly because students did not implement peer marking. The focus group discussions suggested that students’ reactions to participate in peer assessment were negative and concerns were related to the open, transparent nature of the peer assessment tool and the manner in which the assessment was conducted.

Students were mainly worried about the impact that open, transparent peer assessment could have on their relationships with each other. The concept of reciprocity, whereby a fear of reprisal from another team member upon allocation of a lower mark to that individual, was described as one of the primary concerns associated with the current peer marking system [23]. Some teams in the intervention arm reported that marks were fixed at the outset, regardless of individuals’ performance, in an effort to minimise conflict within the group. This is in line with other studies that found that a lack of anonymity in peer assessment can lead to disruption of relations between peers, and teams agreeing they would mark each other positively [31, 32]. Others have therefore suggested that student peer assessment is best conducted anonymously and with clearly defined standardized criteria [15, 21]. However, as mentioned before anonymity can lead to other forms of anxiety and gaming and the challenge of ‘friendly assessment’ is a refractory limitation of peer assessment methods and difficult to circumvent even with the amended assessment approach proposed by participants in this study [23, 33].

Secondly, students expressed concerns about the balanced peer assessment system, where peer marks are fixed and must be achieved by team consensus. This requirement increased students’ anxiety particularly when a fellow student was underperforming. Moreover, the negative approach of our peer assessment system did not allow teams to reward some outstanding members without unfairly having to punish other team member. This finding is in agreement with Levine et al. [34] who found that students in highly functioning teams felt that the points-based peer assessment system unfairly forced them to differentiate. However, in contrast to that study, we did not find that students in more dysfunctional teams felt empowered to score their peers higher or lower based on their performance. In fact, most teams participating in this study elected to assign the same amount of marks to everyone in their team. Finally, novel educational interventions need to bring demonstrable benefits, evaluated using both rigorous randomised trial methodologies and qualitative research, to understand what works, for whom, and when.

### Limitations

There are a number of limitations associated with the design and context of this study. Our undergraduate second-year medical students had no prior experience with peer assessment within the medical school curriculum. This could have resulted in more negative attitudes as has been shown by others [35]. We found that all teams agreed to participate in the research with the hope of avoiding peer assessment. Hostility towards peer assessment is not unique, especially when first experienced [36]. However, others have demonstrated that perceptions do improve once experience is gained in this method of assessment; and a more positive view may appear over time [26, 37].

Second, several teams did not engage in the peer assessment exercise as per the recommended protocol. As previously mentioned, this was partly related to the open, non-anonymous nature of the peer assessment. While students were given some guidance on how to give feedback at the start of the module, the available time-table and staff meant that this was limited in terms of practicing skills in constructive feedback and conflict resolution. Medical schools should introduce peer assessment early in the medical curriculum so that students have the opportunity to develop critical assessment skills and acceptance of being critically evaluated by peers ( [17]. This should preferably be done in a phased approach starting with formative feedback which would be less threatening for students followed by the implementation of (summative) peer marking. As part of this longitudinal approach, students should receive skills training in giving and receiving constructive feedback. Third, given the cultural diversity of RCSI students, intercultural communication challenges could have lead to misinterpretation of students’ activities and reduced their willingness and ability to mark others. Future research should explore whether and how cultural factors play a role in team-based work and peer assessment. Finally, it should be noted that the ultimate benefits of improved skills for evaluating and modifying the performance of one’s peers may not be realised until medical students have graduated and are working in clinical teams, which was the proposition that led to the staff retaining a peer review assessment system over the previous years.

## Conclusions

This study highlights the contribution of mixed-methods research to the development of evidence-based medical education. The findings of the RCT show that this model of peer assessment does not improve team effectiveness or reduce free loading. The qualitative follow-up suggests that likely reasons are the risk or fear of negative consequences for students’ future relationships with their peers, with whom they will be studying for a further 4 years. It is recommended for medical schools to implement less threatening forms of peer assessment and provide guidance and training possibilities for developing critical peer assessment skills early in the medical curriculum.

## Data Availability

The datasets used and/or analysed during the current study are available from the corresponding author on reasonable request.

## Abbreviations

CATME: Comprehensive Assessment Team Member Effectiveness
CONSORT: Consolidated Standards of Reporting Trials
PIH: Population and International Health
RCSI: Royal College of Surgeons in Ireland
RCT: randomised controlled trial
